# Red ginger-extract nanoemulsion modulates high blood pressure in rats by regulating angiotensin-converting enzyme production

**DOI:** 10.14202/vetworld.2021.176-181

**Published:** 2021-01-21

**Authors:** Nada Hanifah, Yusuf Farid Achmad, Aida Humaira, Siti Isrina Oktavia Salasia

**Affiliations:** 1 Department of Clinical Pathology, Faculty of Veterinary Medicine, Universitas Gadjah Mada, Yogyakarta, Indonesia; 2 Faculty of Pharmacy, Universitas Gadjah Mada, Yogyakarta, Indonesia

**Keywords:** angiotensin-converting enzyme, blood pressure, nanoemulsion, red ginger, unilateral ureteral obstruction

## Abstract

**Background and Aim:**

Red ginger (RG) has reportedly been used in folk medicine for the management and prevention of hypertension. One of the hypertension study models in experimental animals is the unilateral ureteral obstruction (UUO). This study aimed at evaluating the effect of RG-extract (RGE) nanoemulsion on UUO-induced hypertension and angiotensin-converting enzyme (ACE) production in rats.

**Materials and Methods:**

RG was extracted using ethanol, combined with virgin coconut oil, polysorbate 80, and polyethylene glycol 400 to form the oil phase. The particle sizes of RGE nanoemulsions were analyzed using a particle size analyzer. The UUO method was used to induce chronic kidney disease in rats (504 mg/200 g and 360 mg/200 g b/w per oral for 7 days). The systolic and diastolic blood pressure was determined non-invasively in conscious state by tail plethysmography using an automated blood pressure monitor. ACE in serum was measured using enzyme-linked immunosorbent assay.

**Results:**

The RGE nanoemulsions exhibited a particle size of32.8 nm and a polydispersity index (PI) of 0.268, indicating a homogenous nanoemulsion. UUO rats treated with RGE nanoemulsion (360 mg/200 g b/w) experienced a significant decrease in both their systolic blood pressure (p<0.05) from 142±1 mmHg to 107±6 mmHg and their diastolic blood pressure from 106±1 mmHg to 84±4 mmHg. Furthermore, treatment with RGE resulted in a 10.80% decrease in the level of ACE.

**Conclusion:**

The size and the PI of the RGE used in this study suggest a stable and effective distribution of the particle size in the emulsions. RGE nanoemulsions at the dose of 360 mg/200 g bw can be used as potential ACE inhibitors because they were found to decrease the blood pressure of hypertensive UUO rats.

## Introduction

Chronic kidney disease (CKD) is a worldwide health problem with a high incidence, prevalence, and mortality rate [1]. CKD is characterized by a progressive kidney decline in kidney function that can lead not only to the emergence of a wide variety of complications, including hypertension, but also to the development of terminal renal failure [2]. Hypertension and CKD are closely interlinked pathophysiologic states, and hypertension can be both a cause and a consequence of CKD. Continuously high systemic blood pressure will eventually disrupt the kidney’s autoregulation system and induce an elevated pressure to the kidneys that injure the kidneys and which can eventually result in the development of CKD [3]. The unilateral ureteral obstruction (UUO) method is a well described model that is used to cause renal fibrosis and CKD [4]. Renin-angiotensin-aldosterone system (RAAS) activation has a significant influence on CKD. One of its prominent components is the angiotensin-converting enzyme (ACE), a glycosylated zinc dipeptidyl-carboxypeptidase whose primary function is to regulate arterial blood pressure and electrolyte balance through the RAAS. Renin breaks down Angiotensinogen to give Angiotensin I (Ang I), and ACE subsequently converts Ang I to Angiotensin II (Ang II).[5]. In the kidneys, the ACE-Ang II type 1 can promote oxidative stress, cell proliferation, sodium, and water retention, inflammation, fibrosis, and vasoconstriction [5]. Oxidative stress is a state of imbalance between excessive oxidant formation and lack of antioxidants as a defense mechanism [6]. In general, there are two types of antioxidants: endogenous and exogenous types. Endogenous antioxidants generally present as enzymatic antioxidants, including superoxide dismutase, catalase, and glutathione peroxidase containing selenium. In contrast, exogenous antioxidants are a type of non-enzymatic antioxidants obtained from daily food intake and which can be classified into hydrophilic (ascorbic acid/Vitamin C, bilirubin, albumin, and flavonoids) and lipophilic (α-tocopherol/Vitamin E, ubiquinol, and carotenoids) [6]. Nonetheless, there are other types of exogenous antioxidants that were previously known as therapeutic agents, such as N-acetylcysteine, bardoxolone methyl, and ACE inhibitors (ACE I) [7,8]. Synthetic ACE I, such as Captopril, is widely used for the treatment of cardiovascular and renal disease [9]. However, the clinical use of this ACE I has been associated with various side effects such as cough, angioneurotic edema, and adverse effects in pregnancy [10,11]. Therefore, it is essential to investigate and identify natural compounds or products that inhibit ACE.

Red ginger (RG) (*Zingiber officinale* Roscoe) contains gingerol and shogaol that could protect the kidney through inhibition of the ACE-I. RG also has an abundant amount of flavonoid, mostly quercetin, which exhibits powerful antioxidant activity [12]. Antioxidants are now used as a therapy to reduce oxidative stress, and RG is believed to influence CKD therapy with its antihypertensive and antioxidant components [13]. RG can be very promising in the pharmacological sector; however, it is classified as a Class II Biopharmaceutical Classification System (BCS) with poor solubility, yet high permeability [14]. To increase its solubility in dosage form, RG is extracted and formulated into nanoemulsion using a water titration method. Water titration or aqueous phase titration is a low energy emulsification method that is commonly used in preparing oil-in-water (O/W) and water-in-oil nanoemulsions [15]. Formulation of drug substances into nanoemulsions has been proved to increase solubility and bioavailability of BCS Class II drugs, such as ezetimibe and anethol trithione [16]. In the water titration method, the drug substance is dissolved in the lipophilic part (oil) of the nanoemulsion; it is then combined with surfactant and co-surfactant, and added with an aqueous phase (water) at a slow rate with gradual stirring [15]. Nanotechnology is a science with many real-life applications including the health and the pharmaceutical fields. Dosage forms with nanoparticles are believed to improve solubility and bioavailability, thus increasing the effectiveness of the therapy [17]. A previously published dissolution study compared the release of ibuprofen nanoemulsion and conventional tablet formulation. It was shown that nanoemulsion exhibited a significantly higher release compared to the tablet formulation, which showed slower release in 1 h [18]. A small globule size in nanoemulsion has a higher surface area that facilitates the faster rate of drug release and increases bioavailability. The emulsion is one of the dosage forms often used in nanotechnology applications because the addition of emulsifiers can decrease the interfacial tension between the water and oil phases and create nano-sized particles [19].

This study aims to investigate at the influence of RG-extract (RGE) nanoemulsion in reducing blood pressure and ACE production in UUO-induced CKD.

## Materials and Methods

### Ethical approval

The experimental protocol was approved by the Animal Ethics Committee for using Animal and Scientific Procedures in the Faculty of Veterinary Medicine, Universitas Gadjah Mada (UGM) with approval number 00.3.5/EC-FKH/int/2019.

### RGE and nanoemulsion formulation

RG was obtained from the Sleman region, Yogyakarta, Indonesia. One part of dried simplisia of RG was mixed with 10 parts of 96% ethanol for 24 h, stirred once every 6 h. The macerate was separated and the process was repeated twice with the same amount of solvent. The entire macerate was then evaporated with a rotary vacuum evaporator to obtain the crude extract. The 0.2 ml of extract was retrieved into a vial and was allowed to react with 5 ml of 75 μM 2,2-diphenyl-1-picrylhydrazyl (DPPH) solution. The mixture was homogenized and remained in a dark place for 30 min. Absorbance was measured using a ultraviolet-visible spectrophotometer at 516 nm. The ability to reduce DPPH radicals (% inhibition), representing the antioxidant activity of the RGE, was calculated using the equation described previously [20,21].

The RGE was formulated into nanoemulsion by a water titration method. The oil phase was a mixture of virgin coconut oil (VCO, Brataco, Indonesia), tween 80 (Brataco) as surfactants, and polyethylene glycol 400 (PEG 400, Brataco) as co-surfactant with ratios of 4:17, 3:8, and 7 [22]. Consequently, 70°C water was added into the oil phase with titration while stirred with a magnetic stirrer until a homogenous nanoemulsion was obtained. The examination of nanoemulsion includes the organoleptic test, pH, stability test, and particle size. Nanoemulsion was observed physically with color, scent, and homogeneity parameters. The acidity levels of the nanoemulsion were observed by measuring the pH with universal pH stick. The particle sizes of nanoemulsion were analyzed using particle size analyzer.

### UUO-induced rats and blood pressure measurement

Sprague Dawley male rats 200 g bw were obtained from the Integrated Research Laboratory UGM, Yogyakarta, Indonesia. All rodents were housed in a constant temperature room, maintained in 12:12 light-dark cycle, and allowed free access to food and water. The UUO is a well-described model of CKD; thus, it was used to induce CKD in rats [4]. The primary advantage of this model is that both kidney injury and fibrosis occur over a time course of days to weeks. Rats were adapted for 1 week before UUO. Briefly, the region undergoing surgery was shaved, and an incision was made on the left side. The rats were anesthetized using ketamine and xylazine intramuscular (i.m.) injection [23]. The left paravertebral area was incised after sterile preparation of the area (2-3 cm) with the scalpel blade to access the left kidney. The ureter was ligated using silk suture at the proximal ureter [24,25]. Following surgery, all rodents were kept in a sterile environment for 10 days.

Twenty-four rats were divided into six groups, namely, P1 (non-UUO rats), P2 (UUO rats), P3 (UUO rats treated with captopril 0.9 mg/200 g b/w, per oral [p.o.]), P4 (UUO rats treated with RGE nanoemulsion at the dose of 540 mg/200 g b/w, p.o.), P5 (UUO rats treated with RGE nanoemulsion at the dose of 360 mg/200 g bw, p.o.), and P6 (UUO rats treated with nanoemulsion base (1.67% v/w, p.o.). Blood pressure parameters, including systolic blood pressure (SBP) and diastolic blood pressure (DBP), were determined non-invasively in conscious state by tail plethysmography using an automated blood pressure monitor (CODA S1, Kent Scientific Corporation, Connecticut, USA).

### ACE measurement

Seven days after treatment ended, approximately 3 ml of blood were collected by retro-orbital venous puncture using plain capillary tubes into plain bottles and allowed to clot. The clotted blood was then centrifuged at 4000 revolution per minute for 10 min and the serum was harvested. ACE in serum was measured using the enzyme-linked immunosorbent assay (ELISA) kit (Wuhan Fine Biotech, China) which is based on sandwich ELISA technology. The anti-ACE antibody was coated onto 48-well plates and the biotin-conjugated anti-ACE antibody was then used for antibodies detection. The standards, test samples, and biotin conjugated detection antibody were subsequently added to the wells and were washed using a wash buffer. Horseradish peroxidase (HRP)-Streptavidin was added, and unbound conjugates were washed away with a wash buffer. Furthermore, 3,3′,5,5′-Tetramethylbenzidine (TMB) substrates were used to visualize HRP enzymatic reactions. This is because TMB is catalyzed by HRP to produce a blue color product that changes into yellow after adding the acidic stop solution. Consequently, the density of the yellow product is proportional to the ACE amount of the sample captured in plate.

### Statistical analysis

Data obtained were analyzed with one-way analysis of variance with a 95% confidence interval. All values were expressed as mean±SD, and the test of significance between two groups was estimated by paired t-tests.

## Results

**Figure-1:**
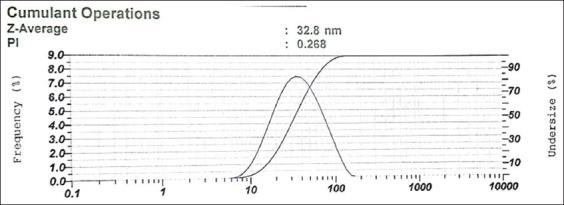
The particle size and polydispersity index of red ginger.

Particle characterization of RGE nanoemulsion: The antioxidant activity test with the DPPH method resulted in a 72% antioxidant activity of RG. Flavonoid test using a spectrophotometer showed 11% w/w flavonoid of RG equivalent with quercetin. To increase its solubility in dosage form, RG was extracted and formulated into nanoemulsion using a water titration method. The appearance of the RGE nanoemulsion was a transparent dark brown, homogenous, with moderate scent of ginger, and a pH level of 5. Figure-1 shows the RGE nanoemulsion particle measured with PSA, the resulted particle size with an average size of about 33 nm, and the polydispersity index (PI) of 0.268.

Blood pressure and ACE measurement: SBP and DBP in UUO rats before and after treatment with the RGE nanoemulsion are shown in Table-1. UUO rats treated with the RGE nanoemulsion at a dose of 540 mg/200 g bw (P4) and 360 mg/200 g bw (P5), respectively, experienced a decrease in SBP (128±3.7 mmHg and 107±6.3, respectively), in comparison to the UUO rats before treatment (136±11 mmHg and 142±1 mmHg, respectively). A decrease in DBP was also identified in UUO rats after treatment with RGE nanoemulsion. The statistical analysis of SBP and DBP with paired sample T-tests revealed a significant decrease (p<0.05) in UUO rats treated with 360 mg/200 g bw (P5).

**Table-1:** The blood pressure of unilateral ureteral obstruction rats.

Groups	Systolic blood pressure (SBP) (mmHg) (x±SE)			Diastolic blood pressure (DBP) (mmHg) (x±SE)		
	
	Pre-RGE	Post-RGE	Sig	Pre-RGE	Post-RGE	Sig
P1	122±5	121±6	0.861	102±4	98±5	0.082
P2	159±8	153±9^ab^	0.506	125±8	118±9^ab^	0.292
P3	165±6	152±2	0.213	131±2	102±5	0.076
P4	136±11	128±4^ac^	0.507	102±13	85±1^ac^	0.332
P5	142±1	107±6^bd^	0.034*	106±1	84±4^bd^	0.031*
P6	159±20	150±2^cd^	0.711	123±19	116±3 ^cd^	0.792

Pre-RGE=Before treated with RGE nanoemulsion, Post-RGE=After treated with RGE nanoemulsion, Sig=Statistical significant difference,

* =p<0.05

**Figure-2:**
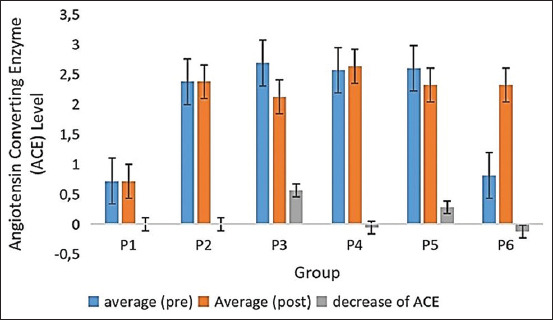
The average of angiotensin-converting enzyme of rat serum.

The measurement of ACE activity in the serum using ELISA revealed a 10.80% decrease in ACE activity of UUO rats after treatment with RGE nanoemulsion at a dose of 360 mg/200 g bw (P5). In contrast, the decrease in ACE activity reached 20.93% in UUO rats treated with Captopril (P3) (Figure-2).

## Discussion

Characteristics of RGE nanoemulsion: The RG extract used in this study revealed an antioxidant activity of 72% and 11% w/w flavonoid of RG equivalent with quercetin, respectively RG has poor solubility yet high permeability.RG was extracted and formulated into nanoemulsion using a water titration method to increase its solubility in dosage form.

Nanoemulsion base used in this study consisted of VCO, polysorbate 80, polyethylene glycol 400, and water. VCO was used as an oil phase because it contains short chain fatty acid, such as lauric acid and myristic acid, which consists of 12 and 14 carbon chains, respectively. Because of this composition of short-chain fatty acid, VCO is slightly hydrophile [26] and it is suitable for use as an oil phase in O/W nanoemulsion. Polysorbate 80 and polyethylene glycol 400 facilitated that the steric stabilization of globules Polysorbate 80 was used as an emulsifying agent due to its suitability for O/W emulsion and low toxicity, and polyethylene glycol 400 was used as a co-surfactant to stabilize the film on nanoemulsion globules. The presence of steric stabilization resulted in the stability of the globule of nanoemulsion. In this research, the RGE nanoemulsion had an average particle size of about 33 nm, which is within the range of small spherical droplets (1-100 nm) [19].

The PI was used to evaluate the degree of non-uniformity of size distribution. This index is dimensionless and scaled, and values smaller than 0.7 are mainly seen in highly monodisperse standards. Conversely, PI values >0.7 suggest that the sample has an extensive particle size distribution. The PI of the nanoemulsion was found to be 0.268, indicating that the particle size distribution is homogenous, and thus an efficient distribution of particle size in the emulsions [27]. In drug delivery applications using lipid-based carriers, a PI value of ≤0.3 is considered acceptable as it indicates a homogenous population of phospholipid vesicles [27]. The size of drug delivery systems is an essential parameter as it can affect pharmacokinetics, tissue distribution, and clearance. Specific physiological processes such as hepatic uptake and accumulation, tissue diffusion, tissue extravasation, and kidney excretion significantly depend on particle size. The aqueous phase titration method used is a low-energy nanoemulsion preparation method, where the nanodroplets are formed through a phase inversion in response to changes in the composition when the aqueous phase is added to the oil phase. This change of composition resulted in the low interfacial tension of the oil-water interface. This method can be achieved through a simple batch stirrer, thus requiring less input energy density [15]. Compared to the high-energy method, the low-energy method offers more efficiency and ease of preparation because of the spontaneous emulsification [28]. A previous study verified that nanoemulsion prepared using the aqueous phase titration method resulted in thermodynamic stability and enhanced oral bioavailability [29].

Effect of RGE nanoemulsion in decreasing of blood pressure and ACE activity: UUO rats treated with the RGE nanoemulsion experienced a decrease in their SBP and DBP. Our findings suggest that RGE nanoemulsions are a very promising method for decreasing blood pressure. The UUO model in mice induces kidney ischemia and hypoxia which lead to interstitial inflammation and tubulointerstitial fibrosis. The initial injury at the acute onset of kidney obstruction in UUO rats induces changes in the glomerular filtration rate, renal blood flow, and interstitial edema [30]. Tubulointerstitial fibrosis can be developed as a result of various kidney injuries and it can cause CKD and hypertension. Based on ACE activity measurements in the serum using ELISA revealed the decreasing of the ACE activity of the UUO rats to a level of 10.80% after treatment with RGE nanoemulsion at a dose of 360 mg/200 g b/w as well as UUO rats treated with captopril. This result indicates that RGE nanoemulsions can inhibit ACE production as well as the ACE I drug of captopril.

Imbalances in the antioxidants levels and in the reactive oxygen species (ROS) that favor ROS in the oxidative stress has been disclaimed in the pathogenesis and pathophysiology of various disorders of cardiovascular disease conditions and hypertension. Reports have shown that oxidative stress is elevated in hypertension, and certain antihypertensives such as the *β*-adrenergic blocker, calcium channel blockers, and ACE I are known to possess antioxidant properties. Therefore, and asides from their primary mechanism of action, they can also target oxidative stress reduction [31]. The previous *in vitro* studies have shown that ginger extract can inhibit ACE (also called ACE-I). The constituent of RG that acts as ACE-I is a flavonoid. RGE may lower blood pressure *in vivo* in rats. RG’s mechanism of reducing blood pressure and the use of flavonoids as ACE-I in regulating blood pressure have both been proven effective in suppressing ACE activity. In addition to being effective as ACE-I, flavonoids also function as antioxidants. RG contains gingerol and shogaol, and thus it can protect kidneys through inhibition of the ACE-I. RG also has an abundant amount of flavonoids, mostly quercetin, as an antioxidant [12]. Antioxidants are now used as a therapy to reduce oxidative stress [13]. RG, with its antihypertensive and antioxidant components, is believed to influence CKD therapy.

## Conclusion

The RGE nanoparticles used in this study were found to have a size of 32.8 nm and a PI 0.268. These measurements suggest that there is a stable and good distribution of the particle size in the emulsions. More importantly, the RGE nanoemulsion at the dose of 360 mg/200 g b/w can reduce blood pressure in UUO-induced hypertension rats because they induce a 10.80% decrease in the level of ACE. Conclusively, RG nanoemulsions containing flavonoids, mostly quercetin, seem to be very promising ACE I.

## Authors’ Contributions

SIOS designed and supervised the research. NH and YFA handled, induced and treated UUO rats. AH extracted and nano-emulsified the RG. SIOS and NH analyzed the data, wrote, and revised the manuscript. All authors read and approved the final manuscript.
